# Re-arthrodesis after primary ankle fusion: 134/1,716 cases from the Swedish Ankle Registry

**DOI:** 10.1080/17453674.2018.1488208

**Published:** 2018-06-27

**Authors:** Anders Henricson, Lars Jehpsson, Åke Carlsson, Björn E Rosengren

**Affiliations:** 1 Department of Orthopedic Surgery, Falun Central Hospital, Falun;; 2 Department of Clinical Sciences and Orthopedic Surgery, Skåne University Hospital, Malmö, Sweden

## Abstract

Background and purpose — Arthrodesis is the most common treatment of severe ankle arthritis. Large studies on the occurrence of re-arthrodesis are few, especially with information in terms of risk. We used the National Swedish Ankle Registry to assess incidence and risk factors for re-arthrodesis.

Patients and methods — In the Registry, we examined the occurrence of re-arthrodesis in 1,716 patients with a primary ankle arthrodesis. We also analyzed associations between the re-arthrodesis risk and sex, diagnosis, and surgical method.

Results — The risk of first re-arthrodesis at 2.5 years was 7.4% and the rate at 9 years 7.8%. The risk following arthroscopic surgery with fixation by screws was 15%, which is statistically significantly higher than the 8% following the gold standard technique with open screw fixation, the 5% following fixation by intramedullary nailing, and the 3% following fixation by plate and screws. Patients with either idiopathic osteoarthritis or posttraumatic arthritis had a higher risk of re-arthrodesis than patients with rheumatoid arthritis. We could not find that the risk of re-arthrodesis was associated with sex.

Interpretation — In Sweden, the re-arthrodesis risk varied by primary technique and was especially high after arthroscopic surgery. Reasons are unknown but poor surgical technique and/or surgeon inexperience may contribute, as may patient selection.

Ankle arthrodesis is the most common surgical treatment of severe ankle arthritis. The most frequent complication after ankle arthrodesis is non-union; the reported rate varies between 0% and 31% (see review articles by Nihal et al. 2008, and Yasui et al. 2016a). Studies concerning different non-union rates related to fixation methods or techniques are sparse.

We examined potential differences in re-arthrodesis risk related to sex, diagnosis, and different surgical methods in a large series of primary ankle fusions followed in the Swedish Ankle Registry.

## Patients and methods

Arthrodesis procedures of the ankle have been reported to the National Swedish Ankle Registry since 2008. The procedure-based coverage is now 96%. The different kinds of fixation and surgical techniques as well as sex, date of birth, date of surgery, and preoperative diagnoses are reported to the registry. Regarding re-arthrodesis the date of secondary surgery and fixation method are registered and also whether a second or third attempt to fuse the ankle has been undertaken.

We identified 1,773 primary ankle arthrodeses from 2008 to 2015. 54 departments had reported between 1 and 203 ankle arthrodeses to the registry; 4 departments reported only 1 arthrodesis each.

45 patients had undergone bilateral ankle arthrodesis. The second primary arthrodesis in these patients was excluded from analysis. 12 patients had less than 6 months’ interval between the primary arthrodesis and the re-arthrodesis and were excluded. Thus 1,716 primary ankle arthrodeses (and patients) were included in this study.

We examined the registry records for any re-arthrodesis procedures until December 2016 and estimated the rate of revision (irrespective of follow-up time) by taking the number of first revisions divided by the number of primary procedures undertaken. At 2.5 years of follow-up 93% of all re-arthrodeses had been undertaken and this time-point was chosen for risk estimation. We also analyzed the association between re-arthrodesis occurrence and sex, diagnosis, and surgical method.

### Statistics

We used chi-square test to compare risk of re-arthrodesis related to sex, different surgical methods, and diagnoses. We considered a p-value <0.05 as statistically significant. Additionally, we undertook a survival analysis (revision-free survival following the primary procedure) of different methods using Kaplan–Meier curves and the log-rank test.

### Funding and potential conflicts of interest

The Swedish Ankle Registry is partly maintained by funds from the Swedish Association of Local Authorities and Regions. The authors received no financial support for the research, authorship, and publication of this article. No conflicts of interest declared.

## Results

Of the 1,716 patients included, 935 were men (median age 50 years [15–87]) and 781 women (median age 53 years [15–91]). There were 540 tibio-talocrural (TTC) primary arthrodeses, 485 (90%) of these performed by retrograde intramedullary nailing. The mean follow-up time was 4.0 years (1.0–9.0).

Posttraumatic arthritis and open screw fixation were the most common diagnosis and treatment, respectively (Table 1). Congenital deformities were the most common entity among “other diagnoses” (Table 2). Information concerning bone grafting was found in 1,295 of the 1,510 cases using open techniques (in 276 cases no bone grafting was used, in 678 harvested from the ankle, in 125 from the tibia, in 100 from the iliac crest, and in 16 allograft was used).

**Table 1.** Distribution of primary arthrodeses by sex, diagnosis, and method of fixation. Values are number of cases

				Diagnosis					
Method of fixation	Total	Men	Women	OA	Post- traumatic arthritis	RA	Diabetes	Neurological disoders	Other
All	1,716	935	781	460	729	213	39	66	209
Open screw fixation	888	496	392	288	430	59	2	21	88
Intramedullary nail	473	249	224	76	145	115	25	37	75
External fixation	48	29	19	6	20	1	7	1	13
Plate and screws	87	53	34	22	37	3	5	5	15
Arthroscopic screws	183	93	90	66	90	11		2	14
Percutaneous screws	23	7	16		3	18			2
Other methods ^a^	14	8	6	2	4	6			2

**^a^**The group of “Other methods” consisted of 5 percutaneous intramedullary retrograde nailings,3 arthroscopic intramedullary retrograde nailings, 1 arthroscopic external fixation, 1 fixation by staples, and 4 with missing data.

**Table 2.** Surgical methods in other diagnoses. Values are number of cases

Methods of fixation	Total	Congenital deformities	Acquired deformities	Post- infectious arthritis	Psoriatic arthritis	Talar osteo- necrosis	Unspecified secondary osteoarthritis	Instability	Hemo- chroma- tosis	Osteo chondritis	Other**^a^**
All	209	36	27	31	14	13	16	10	8	7	47
Open screw fixation	88	13	12	14	5	3	10	6	2	6	17
Intramedullary nail	75	17	14	9	8	5	4	2	4		12
External fixation	13	3	1	1		4					4
Plate and screws	15	3		4		1	1		1	1	4
Arthroscopic screws	13			3	1		1	1	1		6
Percutaneous screws	2										2
Other methods	3							1			2

**^a^** Rare and occasional diagnoses

We identified a re-arthrodesis in 134 of the 1,716 (7.8%) ankles with a primary arthrodesis (Tables 3 and 4). The median time between primary surgery and re-arthrodesis was 1.4 (0.5–6.9) years. 8 (5%) patients underwent a second re-arthrodesis.

**Table 3.** Distribution of re-arthrodeses at 2.5-year follow-up by sex, diagnosis, and method of fixation. Values are number of cases

							Diagnosis	
Method of fixation	Total	Men	Women	OA	Post-traumatic arthritis	RA	Septic arthritis	Other
All	101	59	42	31	52	5	1	12
Open screw fixation	59	34	25	17	32	3		7
Intramedullary nail	19	16	3	5	9		1	4
External fixation	2		2		2			
Plate and screws	2	2		1				1
Arthroscopic screws	18	6	12	8	9	1		
Percutaneous screws	1	1				1		
Other methods	0							

**Table 4.** Total number of arthrodeses and re-arthrodeses, and rate of re-arthrodeses per primary method of fixation

Method of fixation	Patients at risk, n	Patients with re- arthrodesis, n	Re-arthro- desis rate, %	Years of follow-up mean (range)
All	1,716	134	7.8	4.0 (1.0–9.0)
Open screw fixation	888	77	8.6	4.1 (1.0–9.0)
Intramedullary nail	473	25	5.3	4.6 (1.0–9.0)
Plate and screws	87	3	3.4	3.7 (1.0–6.8)
External fixation	48	3	6.3	4.6 (1.2–8.9)
Arthroscopic screws	183	25	14.0	3.5 (1.0–8.8)
Percutaneous screws	23	1	4.3	5.7 (1.6–8.8)
Other methods	14	0	0	4.0 (1.6–8.8)

We found similar rates of re-arthrodesis in men (8.1%) and women (7.4%) (p = 0.7), in cases with (7.2%) and without bone grafting (6.4%) (p = 0.6), and in cases with primary talocrural arthrodesis (TC) (8.6%) and TTC (6.1%) (p = 0.8).

The overall 2.5-year risk of re-arthrodesis was 7.4% but risks differed depending on fixation method. Arthroscopic screw fixation was associated with higher risk compared with open surgery with screws, plate and screws, and retrograde intramedullary nailing (Table 5).

**Table 5.** Patients with primary arthrodesis with follow-up for 2.5 years, risk of re-arthodesis, and RR (risk ratio) for revision (cases) by primary method compared with arthroscopic screws

Method of fixation	Patients with follow-up for 2.5 years	Cases	Non- cases	Risk (%)	Risk ratio (95% CI) ^a^
All	1,385	101	1,284	7.4	
Open screw fixation	716	59	657	8.2	0.55 (0.34–0.91)
Intramedullary nail	407	19	388	4.7	0.31 (0.17–0.58)
Plate and screws	64	2	62	3.1	0.21 (0.05–0.88)
External fixation	42	2	40	4.8	0.32 (0.07–1.3)
Arthroscopic screws	121	18	121	14.9	Ref.
Percutaneous screws	21	1	20	4.8	0.32 (0.05–2.3)
Other methods	14	0	14	0	0

**^a^**Compared with fixation with arthroscopic screws (Ref.).

Survival analysis of primary ankle arthrodesis with re-arthrodesis as end-point showed the lower survival rate of re-arthrodesis for arthroscopic screw fixation compared with open screw fixation, plate and screws, and retrograde intramedullary nailing (log-rank p < 0.001) (Figure, Table 6).

**Table 6.** Life table: number at risk at the beginning of each interval

	Years from surgery							
Method of fixation	0	1	2	3	4	5	6	7
Arthroscopic screws	182	140	96	69	41	23	10	2
Intramedullary nail	467	424	345	264	188	125	84	50
Open screw fixation	884	772	586	424	286	180	107	53
Plate and screws	86	71	55	41	27	14	4	0

In patients with rheumatoid arthritis the rate of re-arthrodesis was 8.6% in those who had undergone TCs and 6.1% in those who had undergone TTCs. We found a higher risk for re-arthrodesis at 2.5 years in patients with idiopathic (RR 3.3 (95% CI 1.3–8.4)) or post-traumatic osteoarthritis (RR 3.3 (1.3–8.2)) than in patients with rheumatoid arthritis (4.2%).


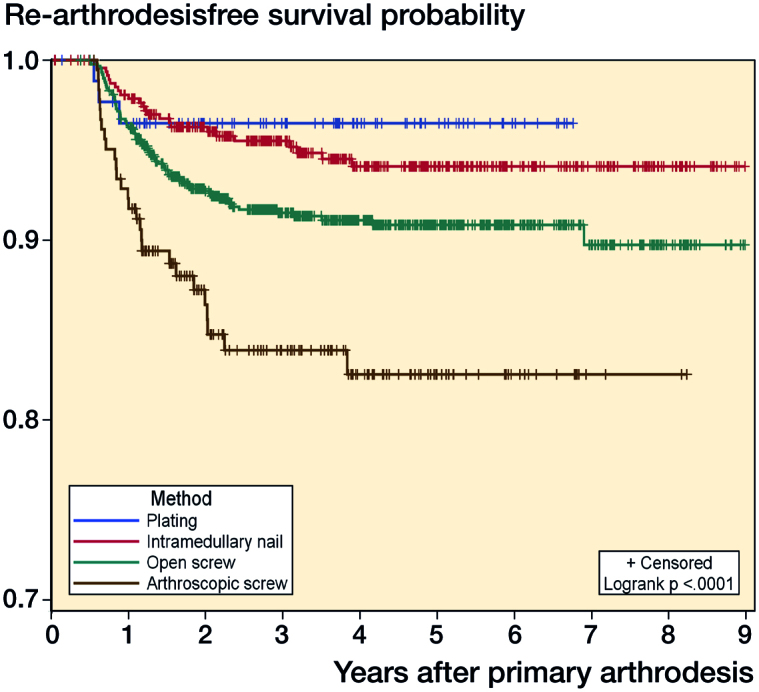


Survival analysis of different methods of fixation.

## Discussion

In this study from the National Swedish Ankle Registry the overall re-arthrodesis risk at 2.5 years was 7.4% but varied depending on primary technique; substantially, arthroscopic ankle arthrodesis with screws was associated with a higher risk for re-arthrodesis than open technique with screws, plate and screws, and retrograde intramedullary nailing.

When comparing the results of screw arthrodesis with open and with arthroscopic technique, Yasui et al. (2016b) in 8,474 patients could not detect any differences between these techniques concerning re-arthrodesis rate. Comparison of arthroscopic screw fixation versus open screw fixation was also studied by Myerson and Quill (1991), Nielsen et al. (2008), and Quayle et al. (2018). They found non-union rates of 2–6% with the arthroscopic technique and of 0–17% with the open technique. Quayle et al. (2018) reported a re-arthrodesis rate of 3% with open technique and 0% with arthroscopic technique.

Re-arthrodesis rates are usually somewhat lower than non-union rates since some patients with a non-union do not need, want, or are unable to undergo reoperation (Collman et al. 2006, Nielsen et al. 2008, Abicht and Roukis 2013, Chayalon et al. 2015, Quayle et al. 2018).

Results of arthroscopic ankle arthrodesis have been evaluated in several reports with follow-up of mean 3 (1–5.5) years and non-union rates of 0–13% in series of 23–104 patients (Saragas 2004, Winson et al. 2005, Collman et al. 2006, Gougoulias et al. 2007, Nielsen et al. 2008, Dannawi et al. 2011, Townshend et al. 2013, Jain et al. 2016, Duan et al. 2017, Kolodziej et al. 2017). A re-arthrodesis rate of 7% was found in 8,474 ankles by Yasui et.al. (2016b). The re-arthrodesis rate varies between other smaller series (Collman et al. 2006, Gougoulias et al. 2007, Nielsen et al. 2008, Dannawi et al. 2011, Townshend et al. 2013, Jain et al. 2016, Kolodziej et al. 2017).

The re-arthrodesis rate (2.5-year risk 15%) for arthroscopic technique was in our study considerably higher than in most of the above-mentioned reports. We speculate that this may partly be because many of these procedures are performed by general foot and ankle surgeons rather than surgeons specially trained in arthroscopic techniques. The 183 arthroscopic ankle fusions in our study were performed by 31 different surgeons and of these 13 surgeons had performed only 1 procedure. We found only 4 surgeons that had performed more than 10 arthroscopic arthrodeses. These findings indicate that arthroscopic ankle fusions are more demanding, have a longer learning curve, and require a higher volume for successful outcome.

The re-arthrodesis rate with open screw technique (2.5-year risk 8%) in our study is in the same range as other studies that report rates of 3–17% in cohorts of 30–49 patients and with the use of 2 or 3 screws for fixation (Maurer et al.^1991^, Moeckel et al. 1991, Nielsen et al. 2008, Townshend et al. 2013). With a 4-screw technique, Zwipp et al. (2010) found a re-arthrodesis rate of only 1 in 72 patients. The Swedish Ankle Registry does not include data on the number of screws that have been used in the open screw technique.

The revision rate (2.5-year risk 3.1%) after plate fixation in our study is consistent with other reports in the literature. Prissel et al. (2017) had 2 non-unions with a single-column anterior plate in 47 patients. Using anterior double plating Plaass et al. (2009) found no non-unions or re-arthrodeses in 29 patients. The Swedish Ankle Registry does not collect data on which plate or how many screws are used in a specific case.

Maurer et al. (1991) and Moeckel et al. (1991) compared the external fixation technique with open screw fixation and found non-union rates of 17% and 21% when external fixation was used and 0% and 5% with the open screw technique and re-arthrodesis rates of 17% and 18% in the external fixation groups. However, these reports are old and the 4.8% revision risk at 2.5 years in our study following external fixation is probably the result of a more modern and refined external fixation technique.

Retrograde intramedullary nailing is often used in more difficult cases with, for example, diabetic arthropathy, neuropathy, large deformities, or rheumatoid arthritis. In our study with 25 re-arthrodeses (5%) out of 473 arthrodesis procedures with intramedullary nailing, half of the patients with re-arthrodesis were in these categories. The 2.5-year risk of revision following fixation with intramedullary nailing was 4.8%. Non-union rates below 10% in series with such diagnoses are reported by Fazal et al. (2006), Chettiar et al. (2011), Lucas y Hernandez et al. (2015), Thomas et al. (2015), Richter and Zech (2016). A 12% non-union rate was found in a study by Pelton et al. (2006). Re-arthrodesis rates of 0–5% were found by Pelton et al. (2006), Fazal et al. (2006), Lucas y Hernandez et al. (2015), Thomas et al. (2015), and Richter and Zech (2016). Fenton et al. (2014) demonstrated many complications with retrograde intramedullary nailing and had 9 non-unions out of 55 ankles; 3 of their 52 patients with postoperative deep infection underwent below-knee amputations. Fazal et al. (2006) also reported 1 below-knee amputation after deep infection. In the present data from the Swedish Ankle Registry no amputations were reported.

Patients with rheumatoid arthritis had an overall lower re-arthrodesis rate than other diagnoses in our study. Reports concerning ankle fusion in rheumatoid arthritis are sparse. The re-arthrodesis rate of 4% in our study (213 primary fusions) is similar to other studies. Fujimori et al. (1999) found no non-unions in 19 patients and Anderson et al. (2005a) found a 4% non-union rate in 26 patients. These studies used retrograde intramedullary nails; in our study more than half of the cases with rheumatoid arthritis underwent this procedure (Table 1). With an open screw technique Anderson et al. (2005b) in 22 rheumatoid patients found non-union in 7 patients and 5 patients had a re-arthrodesis. The authors discouraged this technique in this patient group. In our study the re-arthrodesis rate following open screw fixation was 5% in patients with rheumatoid arthritis.

Diabetes is considered a risk factor for non-union in ankle arthrodesis (Thevendran et al. 2012). Perlman and Thordarson (1999) found a high rate of non-union in diabetic patients while Chalayon et al. (2015) and Jain et al. (2016) were unable to identify any increased risk. In our study there were no re-arthrodeses in the 39 patients with diabetes.

Our study has several limitations. In a registry study, there is always a risk of incomplete reporting. However, the Swedish Ankle Registry has a coverage of 96%. Furthermore, certain details, such as the number of screws and which plates were used, are not presently documented in the registry. The strength is that a registry, especially a nationwide one, covers many procedures, centers, and surgeons of varying experience and proficiency, giving a reasonable depiction of real-life patient care and results.

In summary, in this study from the national Swedish Ankle Registry the 2.5-year re-arthrodesis risk was 7% but varied depending on primary technique. Arthroscopic fusions had a high re-arthrodesis risk (15%). Reasons for this are unknown but poor surgical technique and/or surgeon inexperience may contribute, as may patient selection. Arthroscopic ankle fusion may also be more demanding, have a longer learning curve, and require a higher volume for successful outcome.

Design of study: AH, ÅC, BR, data collection: AH, ÅC, statistics: LJ, data interpretation: all authors revised the manuscript.

*Acta* thanks Markus Knupp and Cees Verheyen reviewers for help with peer review of this study.
